# The first Elcanidae (Orthoptera, Elcanoidea) from the Daohugou fossil bed of northeastern China

**DOI:** 10.3897/zookeys.897.37608

**Published:** 2019-12-09

**Authors:** He Tian, Jun-Jie Gu, Xiang Chu Yin, Dong Ren

**Affiliations:** 1 College of Life Sciences, Capital Normal University, 105 Xisanhuanbeilu, Haidian District, Beijing, 100048, China; 2 Institute of Ecological Agriculture, College of Agronomy, Sichuan Agricultural University, Chengdu, Sichuan, 611130, China; 3 Northwest Institute of Plateau Biology, Chinese Academy of Sciences, 23, Xinning Road, 810008, Xining, China; 4 College of Life Sciences, Hebei University, Baoding, 071002, China

**Keywords:** Archelcaninae, Inner Mongolia, Middle Jurassic, *
Parelcana
*, systematic paleontology, Yanliao Biota

## Abstract

A new species of Elcanidae (Orthoptera, Elcanoidea), *Parelcana pulchmacula***sp. nov.**, is described based on four new specimens from the Middle Jurassic Jiulongshan Formation of northeastern China. The new species differs from all other known Archelcaninae species by its combination of wing-venation characters. This new finding improves our knowledge of variation on wing venation in elcanid insects and constitutes the first record of Elcanidae from the Daohugou fossil bed (Yanliao Biota) of northeastern China.

## Introduction

The extinct family Elcanidae is a cryptic group of Orthoptera insects due to their complex anatomical features. The caeliferan-like wing venation, characteristic of this family, groups them close to Caelifera in cladistic analyses (Béthoux and Nel 2002). However, Elcanidae also shares a long, filiform antennae and exerted ovipositors with the suborder Ensifera. The presence of these contrasting anatomical features makes the systematic position of Elcanidae unclear.

The Elcanidae existed from the Upper Triassic to the Cretaceous in Eurasia and America (Handlirsch 1906; Sharov 1968; Martins-Neto 1991; Gorochov et al. 2006; Poinar et al. 2007; Peñalver and Grimaldi 2010; Fang et al. 2015; Fang et al. 2018a, Fang et al. 2018b; Heads et al. 2018; Tian et al. 2019). So far 50 species in 16 genera have been described from compression fossils and ambers. These species have been divided into two subfamilies, the Archelcaninae and the Elcaninae, based on taxonomic characters (Gorochov et al. 2006). Elcanids evolved a unique character among orthopterans, i.e., the presence of various spurs on the distal part of the metatibia. These structures might have been associated with an improved capability to swim (Tian et al. 2019).

The Jurassic elcanids are well known from the UK (Handlirsch 1906; Whalley 1985), Germany (Handlirsch 1906; Ansorge 1996), Kazakhstan (Sharov 1968), and Kyrgyzstan (Sharov 1968). In China, two specimens have been reported, one from the Guangxi Province (from Early Jurassic), and one from the Hebei Province (from Middle Jurassic). Both were attributed to *Elcana reticulata* (Handlirsch, 1939), based on existing highly fragmented forewing sample specimens (Handlirsch 1939; Hong 1983; Lin 1986). Other Orthoptera are commonly discovered from the Daohugou fossil bed, at the widely known and profuse fossil assemblages of Yanliao Biota, northeastern China. Numerous species discovered from this fossil bed have been described, including ensiferans and caeliferans (Ren and Meng 2006; Ren et al. 2010, 2019; Gu et al. 2009, 2011, 2012, 2016; Wang et al. 2017). In this report we describe a new species of Elcanidae, *Parelcana pulchmacula* sp. nov., from the Daohugou fossil bed. The new species is erected based on four isolated but well-preserved forewings, providing new insights into the complex wing-venation patterns of elcanids.

### Geological setting

**Figure 1.**
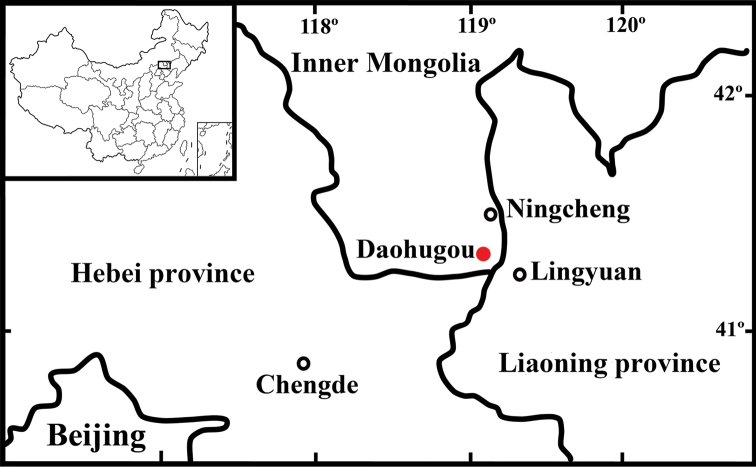
Location map for *Parelcana pulchmacula* sp. nov.

The specimens described here were collected in the Daohugou Bed, located along the boundaries of the provinces of Hebei, Liaoning and Inner Mongolia (Fig. 1). The Daohugou Bed has been previously assigned to the Middle Jurassic Jiulongshan Formation (Ren et al. 2002, 2010, 2019). The Mesozoic section of the Daohugou Bed is mainly composed of tuffaceous conglomerates, tuffaceous siltstones, tuffaceous mudstones, tuffaceous shales, and volcanic breccias. Isotopic radiometric dating of rock samples from the Daohugou area enabled assessment of the age of the Jiulongshan Formation at circa 168–164 million years (Chen et al. 2004; He et al. 2004; Liu et al. 2006; Yang and Li 2008; Chang et al. 2014). This indicates that the age of the Jiulongshan Formation falls within the Bathonian–Callovian boundary interval (Xu et al. 2016).

## Materials and methods

The wing specimens were examined with a Nikon SMZ 25 microscope, and photographed with a Nikon DS-Ri 2 digital camera system. Line drawings were prepared using Adobe Illustrator CC 17.0.0 and Adobe Photoshop CC 14.0 graphics software. The measurements were taken using Adobe Illustrator. The lengths of wings were measured from the apex to the visible base of the wing; the widths of wings refer to the maximum width of the wing. The specimens are deposited in the Key Lab of Insect Evolution & Environmental Changes, Capital Normal University (CNU), Beijing, China.

Wing-venation analyses followed the interpretation proposed by Béthoux and Nel (2001, 2002). Corresponding abbreviations used in taxonomical descriptions are as follows: CP, posterior costa; ScA, ScP, anterior and posterior subcosta, respectively; RA, RP, anterior and posterior radius, respectively; M, media; MA, MP, anterior, posterior media, respectively; CuA, CuP, anterior, posterior cubitus, respectively; CuPaα, the anterior branch of first posterior cubitus; CuPaβ, the posterior branch of first posterior cubitus; CuPb, the second posterior cubitus; AA1, first branch of anterior anal vein.

## Systematic paleontology

### Class Insecta Linnaeus, 1758

#### Order Orthoptera Olivier, 1789

**Suborder Ensifera Chopard, 1920**

**Superfamily Elcanoidea Handlirsch, 1906**

**Family Elcanidae Handlirsch, 1906**

**Subfamily Archelcaninae Gorochov, Jarzembowski & Coram, 2006**

##### Genus Parelcana

Taxon classificationAnimaliaOrthopteraElcanoidea

Handlirsch, 1906

F804743E-896A-583A-BAA0-1D44A185AC5B

###### Type species.

*Parelcana tenuis* Handlirsch, 1906.

###### Composition.

*Parelcana tenuis* Handlirsch, 1906 (Jurassic, Dobbertin, Germany), *P. anglicana* Handlirsch, 1939 (Jurassic, Binton, UK), probably *P. dubia* Handlirsch, 1939 (Jurassic, Gloucester, UK) and *Parelcana pulchmacula* sp. nov. (Handlirsch 1939; Cigliano et al. 2019).

###### Revised diagnosis.

ScP with numerous branches ending at the anterior margin; M with 3 branches before RP fused with MA1; short CuA; CuPaβ, CuPb, and AA1 detached from each other.

###### Comments.

All species of genus *Parelcana* are based on forewing structure. The forewing of *Parelcana* differs from other genera in Archelcaninae by the presence of 3 branches of M before RP fuses with MA1, and presence a very short and nearly vertical CuA.

##### Parelcana pulchmacula sp. nov.

Taxon classificationAnimaliaOrthopteraElcanoidea

C50C6E52-3206-5624-9909-12C1A61ADDB6

http://zoobank.org/8BD8814A-4A53-41C6-8648-00855507D6CF

Fig. 2

###### Diagnosis.

ScP with 6–8 branches ending in anterior margin; CuA very short and fusion with CuPaα before ScA ends in anterior margin, CuA+CuPaα long and S-shaped; occurrence of two big and round dark spots in distal half of wing and one small spot covering the area of CuPa.

###### Etymology.

From the latin ‘Pulch-’ for beautiful and ‘macula’ for patches, referring to the beautiful spots and coloration of the forewing.

###### Type materials.

***Holotype***, CNU-ORT-NN2016041; ***Paratypes***, CNU-ORT-NN2016035; CNU-ORT-NN2016036; CNU-ORT-NN2016042.

###### Locality and age.

Daohugou Village, Shantou Township, Ningcheng County, Inner Mongolia, China; Jiulongshan Formation, Middle Jurassic.

###### General description.

**Figure 2.**
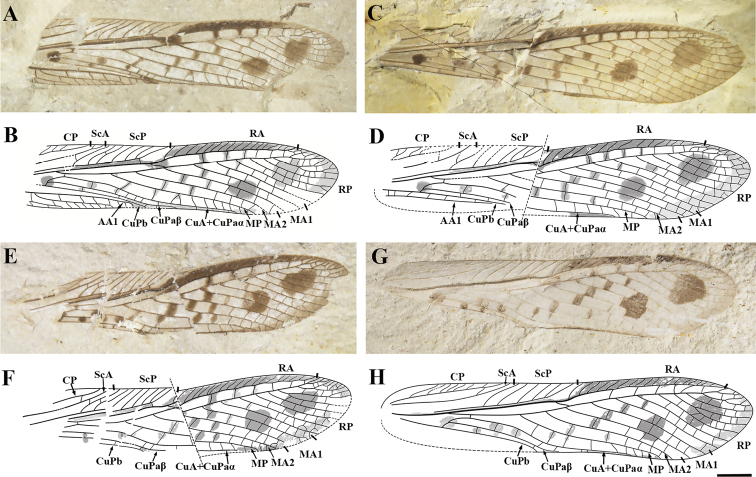
Photo and line drawing of *Parelcana pulchmacula* sp. nov. **A, B** holotype, CNU-ORT-NN2016041 **C, D**CNU-ORT-NN2016035 **E, F**CNU-ORT-NN2016042 **G, H**CNU-ORT-NN2016036. The inclined and dotted lines in the middle of the wings of **D** and **F** represent the cracks in the specimen. The dotted line on the wing venation represents the imaginary line of the wing. Scale bar: 2 mm.

Forewing 18.4–20.9 mm long and 4.3–5.0 mm wide (maximum width recorded). Costal area long and narrow; CP nearly straight, ending in anterior margin after the forking of M+CuA, generating numerous distinct oblique branches ending in the anterior margin; ScA slightly curved, ending in the anterior margin before 1/3 of total wing length; ScP reaching anterior margin at nearly half-length of wing and generating 6–8 oblique branches ending in the anterior margin; stem R+M+CuA forking into R and M+CuA after the divergence point of CuPa; stem R long and distinctly strong, branched into RA and RP near the mid-length of wing; area between ScP and R narrow; RA slightly curved towards posterior wing margin before its first branch, reaching anterior margin close to apex with 16–18 oblique branches; RP with 10–12 comb-like branches reaching wing margin, most of them reaching posterior margin, with several distal terminals dichotomizing and reaching anterior margin; area between RA and RP relatively wide; M forking into MA and MP near to the end of ScA; MA branching into MA1 and MA2 close to the end of ScP; MA1 with 2 branches, with the first fused with RP; MP simple, originates after ScA ends at anterior margin; CuA extremely short, 0.16 to 0.20 mm long, originates before CP ends at anterior margin; CuA almost vertical against the posterior margin; free CuPa short, 0.23 to 0.38 mm long, directed to anterior wing margin, forking into CuPaα and CuPaβ before (Fig. 2A–D) or at the level (Fig. 2G, H) of the bifurcation point of M+CuA; free part of CuPaα approximately three times longer than CuPa, then fused with CuA; CuA+CuPaα simple, long and S-shaped, reaching posterior margin at 2/3 of wing length; CuPaβ simple, similar to CuPaα in shape; CuPb simple; areas between CuPaβ–CuPb and CuPb–AA1 narrow; CuPaβ, CuPb, and AA1 detached each other; AA1 strong and straight; area between branches of RP and M covered with simple and straight crossveins. Dark colorations cover the areas between ScP–R and RA–anterior margin, and also along several rows of the crossveins between branches of RP and M; occurrence of two big and round spots in distal half of wing, one located between the branches of RP, one located at the boundary of RP branches and MA1; one small round spot covers the area of CuPa.

#### Specimen description

**CNU-ORT-NN2016041** (Fig. 2A, B). Holotype, forewing nearly complete with only basal and posterior margin partially missing, 18.4 mm long and 4.3 mm wide (the maximum width, the same below). CP with 3 oblique branches preserved; ScA with 2 branches ending in anterior margin; ScP with 7 branches ending in anterior margin; RA with 17 oblique branches; RP with 10 pectinate branches reaching wing margin; RP fused with anterior branch of MA1 slightly after the ramification point of MA1; CuPaβ reaches the posterior wing margin distally to the end of ScP.

**CNU-ORT-NN2016035** (Fig. 2C, D). Paratype, forewing lost anal region and split into two pieces at about mid-length by an oblique crevice, preserved 20.7 mm long and 4.8 mm wide. CP with 3 oblique branches preserved; ScA with 1 branch connected with CP; ScP with 1 branch connected with ScA and 7 branches ending in anterior margin; RA with 16 oblique branches; RP with 12 pectinate branches reaching wing margin; shortly after origination of posterior branch of MA1, RP fused with anterior branch of MA1.

**CNU-ORT-NN2016042** (Fig. 2E, F). Paratype, forewing lost basal and anal regions, and an oblique crevice split it into two pieces at about mid-length, remaining part 19.1 mm long and 4.5 mm wide. ScA with 1 branch connected with CP; ScP with 8 branches ending in anterior margin; RA with 18 oblique branches; RP with 12 pectinate branches reaching wing margin; RP fused with anterior branch of MA1 at same level as origination of posterior branch of MA1.

**CNU-ORT-NN2016036** (Fig. 2G, H). Paratype, forewing lost anal region, 20.9 mm long and 5 mm wide. CP with 8 oblique branches; ScA with 1 branch connected with CP; ScP with 6 branches ending in anterior margin; RA with 18 oblique branches; RP with 10 pectinate branches reaching wing margin; RP fused with anterior branch of MA1 after the ramification point of MA1; CuPaβ reaches the posterior wing margin basally to the end of ScP.

## Discussion

This new species can be assigned to Archelcaninae by its relatively wide area between RA and RP, and free distal part of CuPaβ, CuPb and AA1. Its simple ScA, the presence of 3 branches of M before RP fuses with MA1 and a very short CuA support assignment to the genus *Parelcana*. *Parelcana pulchmacula* sp. nov. shares with *P. tenuis* a short and vertical CuA, but differs from *P. tenuis* in its larger size, greater number of branches of ScP and RP, a long and S-shaped CuA+CuPaα, a wider area between CuPb and anals, and the coloration pattern of the forewing. The new species is notably different from *P. anglicana* in its greater number of branches of ScP, free and vertical CuA, wider area between CuA+CuPaα and anal region, and fusion pattern of CuA and CuPaα. *Parelcana dubia* was erected based on a fragmentary forewing. It differs from *P. pulchmacula* sp. nov. in having a wider area between RA and RP and the branching pattern of RP. *Parelcana pulchmacula* sp. nov. is also different from the other two known Chinese Jurassic elcanids. It is much larger than the specimen from the Early Jurassic with an estimated wing length of approximately 9.5 mm. The other specimen from the Middle Jurassic of Hebei was originally assigned to *Elcana reticulata* based on an isolated forewing with only the distal half. Most of the diagnostic characters were missing, making comparisons with the new species difficult. Based on the line drawing patterns described for the wing (see Hong 1983, fig. 28), it might be an elcanid, but its generic assignment is questionable.

Variation in forewing size and wing-venation pattern is common in fossil orthopterans and their relatives from the Palaeozoic to the Mesozoic (Prokop and Ren 2007; Gu et al. 2010, 2011, 2014; Béthoux et al. 2012), even between the left and right forewings of the same individual (Gu et al. 2009). For elcanids insects, the documentation on variation of wing venation is scarce since most of the species are described based on limited and often poorly preserved samples. We observed some variation of forewing venation within specimens of *P. pulchmacula* sp. nov.. The number of branches of ScP, RA and RP was not consistent (Fig. 2). Moreover, CuPaβ reaches the posterior wing margin distally to the end of ScP in CNU-ORT-NN2016041(Fig. 2A, B), but basally to the end of ScP in CNU-ORT-NN2016036 (Fig. 2G, H).

**Figure 3.**
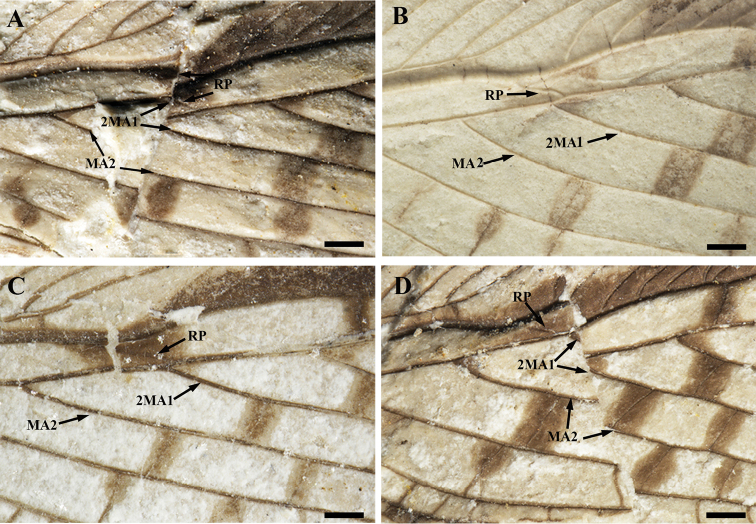
Details of the middle part of the forewings of *Parelcana pulchmacula* sp. nov., showing the variable location of the fusion point of RP and MA1 in the forewing. **A**CNU-ORT-NN2016035 **B**CNU-ORT-NN2016036 **C** holotype, CNU-ORT-NN2016041 **D**CNU-ORT-NN2016042. Scale bar: 0.5 mm.

Based on the known data of wing venation of fossil orthopterans (Gu et al. 2010, 2011, 2014; Béthoux et al. 2012), the differences found in four *P. pulchmacula* sp. nov. specimens should be considered as variations within a species. The location of the fusion point of RP and MA1 is an important character usually used as a distinctive generic character of Elcaninae (Gorochov et al. 2006). In *P. pulchmacula* sp. nov., three of these fusion points show a clear pattern of M with 3 branches before RP is fused with MA1 (Fig. 3A–C). However in specimen CNU-ORT-NN2016042 (Fig. 3D), 2MA1 branches off at the level of RP when reaching MA1. Although this kind of difference always occurs between different species or genus, the integral similarity of wing venation between the four specimens of *P. pulchmacula* sp. nov. indicates that these specimens belong to the same species.

## Supplementary Material

XML Treatment for Parelcana

XML Treatment for Parelcana pulchmacula
